# Investigating the Impact of User Trust on the Adoption and Use of ChatGPT: Survey Analysis

**DOI:** 10.2196/47184

**Published:** 2023-06-14

**Authors:** Avishek Choudhury, Hamid Shamszare

**Affiliations:** 1 Industrial and Management Systems Engineering Benjamin M. Statler College of Engineering and Mineral Resources West Virginia University Morgantown, WV United States

**Keywords:** ChatGPT, trust in AI, artificial intelligence, technology adoption, behavioral intention, chatbot, human factors, trust, adoption, intent, survey, shared accountability, AI policy

## Abstract

**Background:**

ChatGPT (Chat Generative Pre-trained Transformer) has gained popularity for its ability to generate human-like responses. It is essential to note that overreliance or blind trust in ChatGPT, especially in high-stakes decision-making contexts, can have severe consequences. Similarly, lacking trust in the technology can lead to underuse, resulting in missed opportunities.

**Objective:**

This study investigated the impact of users’ trust in ChatGPT on their intent and actual use of the technology. Four hypotheses were tested: (1) users’ intent to use ChatGPT increases with their trust in the technology; (2) the actual use of ChatGPT increases with users’ intent to use the technology; (3) the actual use of ChatGPT increases with users’ trust in the technology; and (4) users’ intent to use ChatGPT can partially mediate the effect of trust in the technology on its actual use.

**Methods:**

This study distributed a web-based survey to adults in the United States who actively use ChatGPT (version 3.5) at least once a month between February 2023 through March 2023. The survey responses were used to develop 2 latent constructs: *Trust* and *Intent to Use*, with *Actual Use* being the outcome variable. The study used partial least squares structural equation modeling to evaluate and test the structural model and hypotheses.

**Results:**

In the study, 607 respondents completed the survey. The primary uses of ChatGPT were for information gathering (n=219, 36.1%), entertainment (n=203, 33.4%), and problem-solving (n=135, 22.2%), with a smaller number using it for health-related queries (n=44, 7.2%) and other activities (n=6, 1%). Our model explained 50.5% and 9.8% of the variance in *Intent to Use* and *Actual Use*, respectively, with path coefficients of 0.711 and 0.221 for *Trust* on *Intent to Use* and *Actual Use*, respectively. The bootstrapped results failed to reject all 4 null hypotheses, with *Trust* having a significant direct effect on both *Intent to Use* (β=0.711, 95% CI 0.656-0.764) and *Actual Use* (β=0.302, 95% CI 0.229-0.374). The indirect effect of *Trust* on *Actual Use*, partially mediated by *Intent to Use*, was also significant (β=0.113, 95% CI 0.001-0.227).

**Conclusions:**

Our results suggest that trust is critical to users’ adoption of ChatGPT. It remains crucial to highlight that ChatGPT was not initially designed for health care applications. Therefore, an overreliance on it for health-related advice could potentially lead to misinformation and subsequent health risks. Efforts must be focused on improving the ChatGPT’s ability to distinguish between queries that it can safely handle and those that should be redirected to human experts (health care professionals). Although risks are associated with excessive trust in artificial intelligence–driven chatbots such as ChatGPT, the potential risks can be reduced by advocating for shared accountability and fostering collaboration between developers, subject matter experts, and human factors researchers.

## Introduction

### Background

Artificial Intelligence (AI) has been a subject of research and intrigue for scientists, engineers, and thinkers since the emergence of computing machines. The genesis of AI can be traced back to the 1950s, marking the commencement of an extensive voyage that would ultimately lead to the development of intricate, human-like machines capable of independent thinking, learning, and reasoning [1]. Initially, AI was perceived as a solution to all problems—a technology that could mechanize every task and supplant human labor. Early research focused on building rule-based systems that could make decisions based on predetermined logical rules. Nevertheless, these systems had limited usefulness as they were rigid and could not learn from data or adapt to novel situations [2]. In the 1960s and 1970s, the emphasis of AI research shifted toward developing expert systems that could reason and make decisions based on extensive domain-specific knowledge [3]. These systems were widely used in various fields, such as medicine, finance, and engineering, and were seen as a major advancement in AI research [4]. However, the limitations of expert systems became apparent in the 1980s and 1990s, as they could not handle the complexity and ambiguity of real-world problems [5]. This led to the development of machine learning algorithms that could learn from data and make decisions based on statistical patterns. With the advent of the internet and the availability of massive amounts of data, deep learning algorithms emerged, which are capable of learning complex patterns in images, speech, and text.

In recent years, AI has been widely adopted in various fields, including health care, finance, transportation, and entertainment. AI-powered technologies such as self-driving cars, virtual assistants, and personalized recommendations have become integral to our daily lives. One of the most substantial breakthroughs in AI research has been the emergence of large-scale language models that are built on Generative Pre-trained Transformers such as ChatGPT (Chat Generative Pre-trained Transformer; OpenAI) [6]. These models are trained on vast amounts of textual data and can generate human-like responses to natural language queries. ChatGPT has revolutionized the field of natural language processing and has paved the way for a new generation of AI-powered language applications. ChatGPT is a cutting-edge language model that OpenAI developed in 2019. It is based on a transformer architecture—a deep learning model that has demonstrated remarkable efficacy in processing sequential data, particularly natural language. ChatGPT was trained on a colossal corpus of text data, which included various sources such as books, articles, and websites.

ChatGPT has garnered substantial traction among computer users, largely due to its impressive ability to generate responses that resemble those of the human language [7-10]. Many users appreciate the convenience and efficiency of this technology, particularly in various applications such as chatbots, virtual assistants, and customer service agents [11-14]. However, along with its burgeoning popularity, ChatGPT has prompted concerns about the broader implications of its use [15-19]. Among these concerns is the potential for its exploitation for malicious purposes, such as social engineering attacks or other forms of fraud [20]. Another issue relates to the possibility of the technology exacerbating preexisting societal biases, as the model’s training data may have inadvertently reflected these biases and cause ChatGPT to produce biased responses [21]. Moreover, ChatGPT’s ability to produce highly convincing fake text has sparked unease regarding its potential misuse in disinformation campaigns, deep fakes, and other malicious activities [22]. These concerns have catalyzed efforts by researchers and policy makers to identify and address the risks associated with this technology, including developing techniques to detect and prevent malicious use and ensuring that the training data used for ChatGPT and similar models are diverse, representative, and free of any biases [22]. Therefore, it is crucial to remain vigilant and proactively address the possible risks arising from its use [23].

The consequences of overreliance or exhibiting blind trust in ChatGPT, particularly in high-stakes decision-making contexts, cannot be overstated. Although impressive in its capabilities, the technology is not impervious to errors, especially if it has been trained on biased or incomplete data. Given its nature of continuously learning from internet texts, failure to adequately verify and validate ChatGPT’s responses can result in incorrect or incomplete decisions, which can have substantial and far-reaching implications in health care, finance, and law [24]. Conversely, a complete lack of trust in ChatGPT can lead to the underuse of this technology. Such distrust can lead to hesitancy to use the technology for decision-making, leading to missed opportunities and slower decision-making processes.

**Figure 1:**
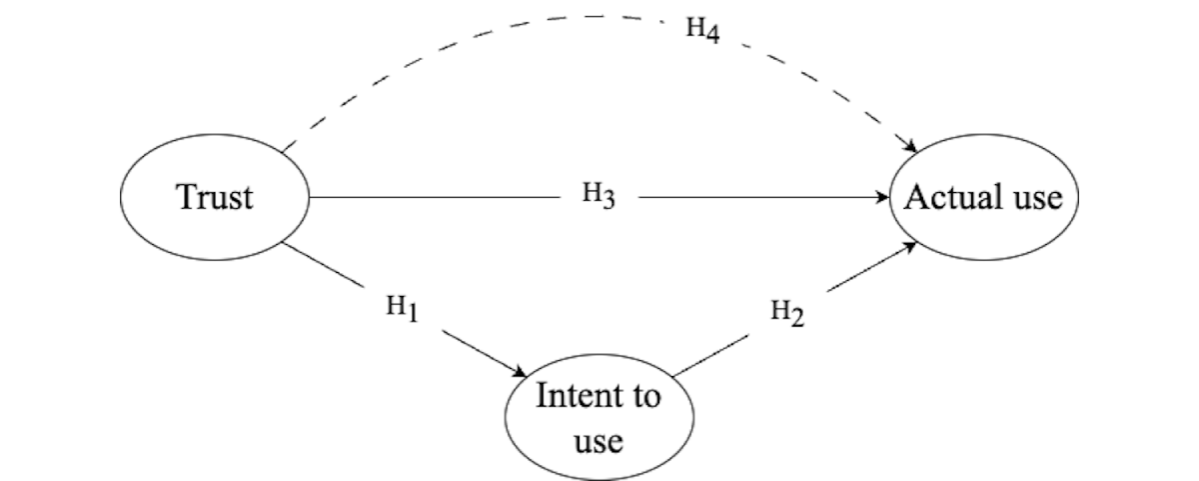
The conceptual structural framework. H1 through H4 indicate the hypotheses. The dashed line connecting trust and actual use indicates the indirect effect, whereas solid lines indicate the direct paths.

Excessive or lack of trust in ChatGPT can have deleterious effects. Striking a balance between trust and validation is essential to ensure the responsible and efficacious use of ChatGPT to maximize its benefits and mitigate its associated risks. Therefore, this study captured users’ trust in ChatGPT and explored its impact on user intent to use the technology. Additionally, it explored its direct and indirect effects on the actual use of ChatGPT. As illustrated in Figure 1, we explored the following 4 hypotheses:

- H1: User’s intent to use ChatGPT increases with their trust in the technology.
- H2: The actual use of ChatGPT increases with users’ intent to use the technology.
- H3: The actual use of ChatGPT increases with users’ trust in the technology.
- H4: Users’ intent to use ChatGPT can partially mediate the effect of trust in the technology on its actual use.

### Hypothesis Development

In this study, we define *Trust* in ChatGPT as a user’s willingness to take chances based on the recommendations made by this technology. This implies that the user believes that the technology has the capacity to execute a particular task accurately while keeping in mind the possibility of negative outcomes. The *Intent to Use* [25] ChatGPT refers to the degree to which an end user perceives the technology as useful and user-friendly and their willingness to adopt and use it for decision-making purposes. *Actual Use* of ChatGPT refers to the extent to which end users have used the technology for decision-making purposes in their respective fields. The extant literature attests to a positive correlation between users’ trust in technology and their inclination to use it, as evidenced by many studies [25-31]. Notably, one investigation probing patients’ and clinicians’ perceptions of chatbots found a substantial nexus between users’ trust in AI-based health care chatbot services and their intention to use them [30]. Similarly, a study examining virtual assistants in the health care domain revealed a positive correlation between users’ trust in the technology and their willingness to use it for managing their health [32]. Furthermore, a study conducted in the marketing realm concluded that chatbots augment customers’ trust and purchase intention [29]. Against this backdrop, we posited that the degree of users’ intent to use ChatGPT will increase concomitantly with their trust in the technology, thereby underscoring a positive association between the 2 variables. We articulated this hypothesis as *H1: users’ intent to use ChatGPT increases with their trust in the technology.*

Successful technology implementation depends on users’ intention to use it and their actual use. Despite users’ intentions to use technology, they may not put it into practice for several reasons, such as the lack of time, resources, technical skills, or negative experiences with the technology [33]. Prior research has established a positive correlation between intent to use and actual use of technology, indicating that users who intend to use the technology are more likely to actually use it [25,31,34]. For instance, studies on adopting robots as assistive social agents found that users’ intent to use them strongly predicted their actual use [25,31]. In addition, research on adopting conversational agents in the form of chatbots for disease diagnosis showed that users’ intention to use the chatbot influenced their actual use of the chatbot [34]. Thus, we hypothesized that users’ intent to use ChatGPT will positively influence their actual use of the technology. We articulated this hypothesis as *H2: the actual use of ChatGPT increases with users’ intent to use the technology.*

Trust can also influence the actual use of ChatGPT. A survey study involving 359 participants revealed that users’ intentions to continue using chatbot services were influenced mainly by their trust in the chatbot [35]. A health care study using interviews revealed that trust is vital in determining whether individuals will use chatbots for disease diagnosis [34]. Specifically, the level of trust in chatbots as conversational agents was a decisive factor in the interviewees’ decision to use the technology. This finding supports the notion that trust positively impacts the actual use of technology, highlighting its critical role in adopting and implementing new technological solutions. Therefore, we hypothesized that trust in ChatGPT will impact the actual use of the technology. We articulated this hypothesis as *H3: the actual use of ChatGPT increases with users’ trust in the technology.*

We also explored the following hypothesis: *H4: users’ intent to use ChatGPT can partially mediate the effect of trust in the technology on its actual use.* If users trust ChatGPT, they may be more likely to form positive attitudes toward using the technology and develop an intention to use it. This intention, in turn, may lead to the actual use of the technology. Therefore, users’ intent to use ChatGPT could be a pathway through which trust in the technology can partially mediate its effect on actual use. A study on technology acceptance for assistive social robots among older adult users found that the intention to use plays a mediating role in the relationship between trust and actual use [31]. This suggests that trust alone may not be sufficient to predict the actual use of assistive social robots among older adult users, as the intention to use plays an important role in this relationship. By considering this potential mediating effect, researchers can gain a more comprehensive understanding of the factors influencing users’ adoption of ChatGPT.

## Methods

### Ethics Approval

The study obtained ethical approval from West Virginia University, Morgantown (protocol 2302725983).

### Semistructured Survey

We distributed a web-based semistructured survey to adults in the United States who actively use ChatGPT (version 3.5) at least once a month. We collected the data from February 2023 through March 2023. The survey was designed on Qualtrics (Qualtrics LLC) and was distributed by Centiment (Centiment LLC), an audience-paneling service. We leveraged Centiment’s service as they reach a broader and more representative audience via their network and social media. They also use fingerprinting technology that combines IP address, device type, screen size, and cookies to ensure that only unique panelists enter the survey.

We conducted a soft launch of the survey and collected 40 responses. A soft launch is a small-scale test of a survey before it is distributed to a larger audience. A soft launch aims to identify any potential issues with the survey, such as unclear or confusing questions, technical glitches, or other problems that may affect the quality of the data collected. The survey was then distributed to a larger audience.

Table 1 shows the descriptive statistics of the survey questions used in this study. We developed 2 latent constructs based on the question (predictors): *Trust* and *Intent to Use*. Participant responses to all the questions were captured using a 4-point Likert scale ranging from 1=*strongly disagree* to 4=*strongly agree*. The *Actual Use* factor, the outcome variable, was captured using a single-item question capturing the frequency of use ranging from 1=*once a month* to 4=*almost every day*.

**Table 1:** Descriptive statistics of study variables (N=607).

Survey items		Value, mean (SD)
**Trust**		
	ChatGPT^a^ is competent in providing the information and guidance I need	3.20 (0.83)
	ChatGPT is reliable in providing consistent and dependable information	3.16 (0.80)
	ChatGPT is transparent	3.12 (0.86)
	ChatGPT is trustworthy in the sense that it is dependable and credible	3.17 (0.84)
	ChatGPT will not cause harm, manipulate its responses, create negative consequences for me	3.10 (0.88)
	ChatGPT will act with integrity and be honest with me	3.19 (0.82)
	ChatGPT is secure and protects my privacy and confidential information	3.27 (0.81)
**Intent to Use**		
	I am willing to use ChatGPT for healthcare related queries	3.10 (0.86)
	I am willing to take decisions based on the recommendations provided by ChatGPT	3.13 (0.82)
	I am willing to use ChatGPT in future	3.38 (0.76)
**Actual Use**		
	How frequently do you use ChatGPT	3.33 (1.10)

^a^ChatGPT: Chat Generative Pre-trained Transformer.

### Statistical Analysis

All the analyses were done in R (R Foundation for Statistical Computing) [36] using the *seminr* package [37]. We evaluated and validated the latent constructs’ convergent and discriminant validity. The convergent and reliability were assessed using 3 criteria [38]: factor loadings (>0.50), composite reliability (>0.70), and average variance extracted (>0.50). The discriminant validity was accessed using the Heterotrait-Monotrait ratio (<0.90) [39]. After validating the latent construct (measurement model), we leveraged the partial least squares structural equation modeling (PLS-SEM) to test the structural model and hypotheses. The PLS-SEM method is a well-established method for multivariate analysis [40]. It allows for estimating complex models with several constructs, indicator variables, and structural paths without imposing distributional assumptions on the data [41]. PLS-SEM is also suitable for small sample sizes when models comprise many constructs and items [42]. Thus, PLS-SEM is a good method for exploratory research as it offers the flexibility needed for the interplay between theory and data [43].

## Results

In all, 607 respondents completed the survey, of which 182 (30%) used ChatGPT at least once a month, 158 (26%) used it once per week, 149 (24.5%) used it more than once per week, and 118 (19.4%) used it almost every day. Most respondents had at minimum a high school diploma (n=204, 33.6%) or a bachelor’s degree (n=262, 43.2%). Most of the respondents used ChatGPT for information gathering (n=219, 36.1%), entertainment (n=203, 33.4%), and problem-solving (n=135, 22.2%). We also noted users who used the technology for health-related queries (n=44, 7.2%) and other activities (n=6, 1%), such as generating ideas, grammar checks, and writing blog content. Participants acknowledged the ease of use, usefulness, and accessibility as the 3 most important factors encouraging them to use ChatGPT. Other factors were in the following order: trustworthiness, algorithm quality, privacy, brand value, and transparency.

Table 2 depicts that the effect of *Trust* on *Intent to Use* was stronger than its effect on *Actual Use*, with path coefficients of 0.711 and 0.221, respectively. The model explained 50.5% and 9.8% of the variance in Intent to Use and Actual Use, respectively. Reliability estimates indicated high levels of internal consistency for all 3 latent variables, with Cronbach α and rho values exceeding the recommended threshold of 0.7. The average variance extracted for *Trust* and *Intent to Use* also exceeded the recommended threshold of 0.5, indicating that these variables are well-defined and reliable. Table 3 shows the Heterotrait-Monotrait ratios for the paths between *Trust* and *Intent to Use*, *Trust* and *Actual Use*, and Intent to Use and Actual Use. The results suggest that the Heterotrait-Monotrait ratios are below the recommended threshold of 0.9, indicating discriminant validity in the model.

**Figure 2:**
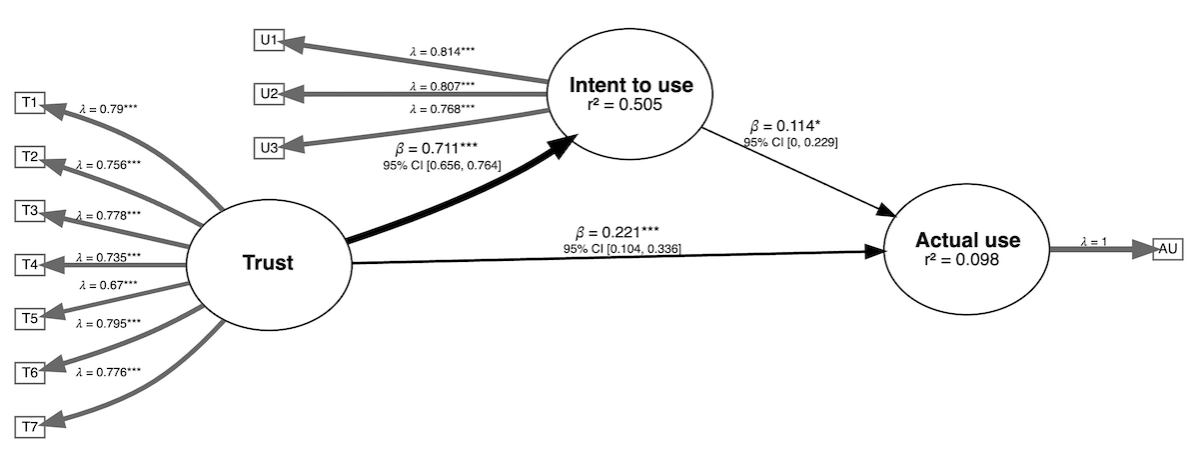
Conceptual framework illustrating the significant paths connecting trust in ChatGPT (Chat Generative Pre-trained Transformer), users' intent to use ChatGPT, and its actual use (AU). T1 through T7: factors for trust; U1 through U3: factors for intent to use. *P<.05 and ***P<.001.

According to our bootstrapped PLS-SEM results, we found support for all 4 hypotheses. Figure 2 illustrates the conceptual framework that connects trust in ChatGPT, users’ intent to use ChatGPT, and its actual use. Factors T1 through T7 indicate the 7 observed variables forming the latent construct of *Trust*, and factors U1 through U3 form the construct of *Intent to Use*. The thickness of the arrows in the inner model reflects the magnitude of the direct effects.

H1 posited that trust in ChatGPT would have a direct effect on users’ intentions to use the technology. Our results confirmed this hypothesis (β=0.711, 95% CI 0.656-0.764), indicating a strong positive relationship.

H2 suggested that users’ intent to use ChatGPT would have an effect on their actual use. This was also supported by our data (β=0.114, 95% CI 0.001-0.229), underlining the role of intent as a predictor of use.

H3 proposed that trust in ChatGPT would directly influence its actual use. Our results corroborated this hypothesis (β=0.302, 95% CI 0.229-0.374), affirming that trust can directly drive actual use.

Finally, H4 postulated that the effect of trust on actual use would be partially mediated by the intent to use. Our analysis also confirmed this, with the indirect effect of trust on actual use through intent to use being significant (β=0.113, 95% CI 0.003-0.227).

**Table 2:** Model fit and reliability measures.

		Intent to Use	Actual Use
**Model fit**			
	*R^2^*	0.505	0.098
	Adjusted *R*^2^	0.504	0.095
	Trust	0.711	0.221
	Intent to Use	N/A^a^	0.114
**Reliability measures**			
	Cronbach α	.876	N/A
	Rho C	0.904	N/A
	AVE^b^	0.575	N/A
	Rho A	0.880	N/A

^a^N/A: not applicable.

^b^AVE: average variance extracted.

**Table 3:** Discriminant validity measures.

	Original estimate	Bootstrap, mean (SD)	95% CI
Trust → Intent to Use	0.896	0.897 (0.035)	0.827-0.962
Trust → Actual Use	0.320	0.320 (0.040)	0.241-0.397
Intent to Use → Actual Use	0.320	0.321 (0.044)	0.233-0.406

## Discussion

### Principal Findings

This is the first study exploring the role of trust in ChatGPT’s adoption from a human factors viewpoint. This study contributes to the extant literature by shedding light on the importance of trust as a determinant of both the intention to use and the actual use of chatbot technologies. Furthermore, the study highlights the mediating role of intention to use in the relationship between trust and actual use. These insights are particularly relevant for organizations and developers seeking to design and market chatbot technologies that users are more likely to adopt and use. Moreover, the results show how users engage with chatbot technologies, including information gathering, entertainment, problem-solving, and health-related queries. This highlights the potential of chatbot technologies to meet various needs and suggests that developers may consider designing chatbots with diverse functionalities to enhance user satisfaction and engagement.

Our findings complement and build upon the insights from the other studies by providing a nuanced understanding of the role of trust in chatbot adoption. Our study found that trust has a significant direct effect on both intentions to use (β=0.711) and actual use (β=0.302) of the technology. Moreover, the indirect effect of trust on actual use, partially mediated by intent to use, was also significant. This aligns with the prior study [44], which explored the antecedents and consequences of chatbot initial trust. They revealed that compatibility, perceived ease of use, and social influence significantly boost users’ initial trust toward chatbots, enhancing the intention to use chatbots and encouraging engagement. Another study [45] focused on the impact of anthropomorphism on user response to chatbots from the perspective of trust and relationship norms. Their findings complement our study by highlighting the role of anthropomorphism in trust formation, ultimately influencing chatbot use. Following the technology acceptance model and diffusion of innovations theory, a prior study [28] examined the intention of users to use chatbots on smartphones for shopping. The study found that attitude toward chatbots was considerably influenced by perceived usefulness, the ease of use, enjoyment, price consciousness, perceived risk, and personal innovativeness. On the other hand, the intention to use was directly influenced only by trust, personal innovativeness, and attitude. Therefore, the study supports our findings by emphasizing the role of trust in the intention to use chatbots and adding other factors such as personal innovativeness and attitude. Similarly, a study [29] reported that credibility, competence, anthropomorphism, social presence, and informativeness influence user trust in chatbots, affecting purchase intention—thus, emphasizing the importance of trust and its antecedents in determining the use of chatbots.

### Theoretical Contribution

Our study makes several important theoretical contributions to understanding trust and its role in adopting and using AI-based chatbots (ChatGPT). By examining the direct and indirect effects of trust on intentions to use and actual use of the technology, the study confirms the importance of trust in the adoption process. It extends the existing literature by highlighting the underlying mechanisms through which trust influences actual use. This new understanding contributes to developing a more comprehensive theoretical framework for studying chatbot adoption.

Our findings emphasize the critical role of trust in adopting and using chatbots. By demonstrating that trust has a significant direct effect on intentions to use and actual use, the study reinforces the centrality of trust in technology adoption research. This is consistent with the findings of prior literature, which also underscore the importance of trust in various aspects of chatbot adoption, such as initial trust [44], response to anthropomorphic attributes [45], and purchase intention [29].

Our study extends the existing literature by uncovering the mediating role of intention to use in the relationship between trust and actual use. By showing that the indirect effect of trust on actual use is partially mediated by intention to use, the study provides valuable insights into the mechanisms through which trust influences actual use. This novel contribution enhances our understanding of the complex interplay between trust and behavioral outcomes, laying the groundwork for future research on the dynamics of trust in technology adoption.

### Policy Implications

Our study’s findings can significantly inform the decision-making processes for policy makers and public administrators as they face the challenges of implementing AI-driven solutions. By emphasizing the importance of trust, our study lays the groundwork for addressing potential pitfalls and governance challenges, ultimately promoting the successful integration of chatbots.

First, establishing trust in AI-powered conversational agents should be a priority for policy makers and technology developers. This can be achieved through transparent disclosure of the agents’ operational processes, information sources, and guiding algorithms. Disclosures should be easily accessible, user-friendly, and presented in clear language. Additionally, conversational agents should include explicit disclaimers to minimize the risk of misleading or erroneous responses.

Second, developers and policy makers should design conversational agents prioritizing user needs and preferences. Incorporating features that allow users to tailor the agent’s responses to their specific requirements, such as tone, vocabulary, and response time, will enhance user satisfaction. Furthermore, agents should prioritize providing accurate and relevant information while minimizing the potential for algorithmic bias, which could result in discriminatory or inaccurate responses.

Third, policy makers should encourage shared accountability to promote the responsible development and deployment of chatbots such as ChatGPT. We define shared accountability as a collaborative approach to ensuring the responsible development and deployment of AI-based technologies, involving stakeholders who share responsibility for ensuring the technology’s accuracy, safety, and ethical use. This approach fosters a culture of transparency and responsibility, enabling stakeholders to identify and address potential issues and optimize the technology for the benefit of all users.

By promoting shared accountability, policy makers can help create a culture of responsibility and transparency that motivates all stakeholders to optimize the technology. For example, developers and data-quality teams will be motivated to ensure that the AI is accurate and reliable. At the same time, users will be encouraged to provide feedback and report any issues or concerns. This sense of accountability and responsibility can make a substantial difference in ensuring that the technology is developed and deployed in a responsible and ethical manner. Furthermore, shared accountability can help to address concerns around biases and other ethical considerations in AI development. By involving diverse stakeholders in the development process, policy makers can ensure that the technology is designed to meet the needs and expectations of a broad range of users while minimizing the risk of unintentional harm or bias.

Lastly, policy makers should establish policies and regulations promoting the responsible development and deployment of conversational agents [46]. These policies should mandate adherence to ethical and legal guidelines related to privacy, data security, and bias. Policy makers should also provide guidance on appropriate use cases for conversational agents, such as information retrieval and customer service. Implementing such policies and regulations will ensure that conversational agents are developed and deployed to maximize benefits while minimizing potential risks and misuse.

### Practical Implications

Our study also contributes to the human factors and health sciences literature by examining the role of trust in adopting AI-driven chatbots such as ChatGPT for health-related purposes. Our findings align with and extend the current understanding of other studies by identifying key factors influencing user adoption, such as trustworthiness, algorithm quality, privacy, transparency, and brand value [47-50]. From a human factors perspective, our study emphasizes the importance of designing chatbot technologies that cater to user needs and preferences while addressing potential concerns and risks.

Moreover, given the increasing use of AI-powered chatbots for various activities, it is important to note that many respondents used the technology for health-related queries. This implies that health providers can leverage chatbots to provide health information and support to patients [8,51,52]. However, to ensure user safety and the accuracy of health information provided, health providers must collaborate with technology providers to develop and integrate reliable and trustworthy health-related information sources into the chatbots [22,53]. Given the complexity and sensitivity of health-related issues, users must exercise caution when seeking health advice from an AI chatbot such as ChatGPT. Users should be aware of the limitations of AI technology in the medical field and should not use ChatGPT as a replacement for professional medical advice. To mitigate these risks, it may be useful for ChatGPT developers to provide clear disclaimers and warnings regarding the limitations of the technology in the medical field and simultaneously work toward integrating reliable medical databases to provide more accurate and trustworthy health advice.

Although risks are associated with excessive trust in AI-driven chatbots such as ChatGPT, it is important to recognize that these technologies continually evolve as they process new data from the internet. However, biased or false information across the web can potentially influence ChatGPT’s responses, reinforcing misinformation or perpetuating skew perspectives. To address this concern, a proactive approach should be gradually adopted to develop mechanisms that filter out false or biased information from the chatbot’s training model.

Since data floating on the internet can be manipulated, systematic efforts should be made to design and implement robust algorithms that identify and remove unreliable or unbalanced data, ensuring that ChatGPT is trained on diverse and accurate information. This can help prevent the chatbot from placing excessive weightage on certain polarities of data, which may result from skewed information on the internet. By refining the chatbot’s training model and incorporating more reliable data sources, the performance of ChatGPT can be continually improved to provide more accurate and unbiased responses.

In addition to these technological improvements, collaboration between developers, subject matter experts, and human factors researchers can further ensure that AI-driven chatbots such as ChatGPT are designed and deployed with a comprehensive understanding of user needs and potential challenges. By addressing the risks associated with excessive trust and actively improving the chatbot’s performance, the development and application of AI-driven technologies such as ChatGPT can continue advancing, promoting positive outcomes and responsible use in various domains.

### Limitations

Our study has limitations, including using a cross-sectional survey and self-report measures, which may introduce biases. The limited geographic scope of the sample, focused on US respondents, may affect the generalizability of our findings to other cultural contexts. Future research should use longitudinal data; explore trust in chatbot adoption across different cultural contexts; and control for potential confounding factors such as participants’ familiarity with AI technology, prior experiences with chatbots, and demographic factors. Future research should use various methods, such as tracking actual chatbot use and conducting qualitative interviews, to assess trust and user behavior. Increasing data collection frequency and ensuring participants’ anonymity can also help mitigate biases. Future research can better understand trust’s role in chatbot adoption by addressing these limitations and enabling developers and organizations to design technologies that meet users’ needs and expectations.

### Conclusion

Our study provides novel insights into the factors driving the adoption of chatbot technologies such as ChatGPT. Our results suggest that trust is critical to users’ adoption of ChatGPT and few people tend to use it for health-related queries. Even as ChatGPT evolves, it remains crucial to highlight that this tool, while powerful, was not initially designed with a specific focus on health care applications. Therefore, an overreliance on it for health-related advice or diagnoses could potentially lead to misinformation and subsequent health risks.

Efforts must also be focused on improving the system’s ability to distinguish between queries that it can safely handle and those that should be redirected to a human health care professional.

Companies and policy makers should prioritize building trust and transparency in developing and deploying chatbots. Although risks are associated with excessive trust in AI-driven chatbots such as ChatGPT, it is important to recognize that the potential risks can be reduced by advocating for shared accountability and fostering collaboration between developers, subject matter experts (such as health care professionals), and human factors researchers.

A systematic collaborative approach can ensure that AI-driven chatbots are designed and deployed with a comprehensive understanding of user needs and potential challenges. By addressing the risks associated with excessive trust and actively improving the chatbot’s performance, the development and application of AI-driven technologies such as ChatGPT can continue advancing, promoting positive outcomes and responsible use in various domains.

## Abbreviations

AI: artificial intelligence
ChatGPT: Chat Generative Pre-trained Transformer
PLS-SEM: partial least squares structural equation modeling
